# Sulfate-reducing bioreactors subjected to high sulfate loading rate or acidity: variations in microbial consortia

**DOI:** 10.1186/s13568-022-01438-2

**Published:** 2022-07-16

**Authors:** Marja Salo, Malin Bomberg

**Affiliations:** VTT Technical Research Centre of Finland Ltd, P.O.Box 1000, 02044 Espoo, Finland

**Keywords:** Biological sulfate reduction, Bioreactor, Sulfate loading rate, Acidity, Microbial consortia

## Abstract

Sulfate-reducing bioreactors are used in e.g. the mining industry to remove sulfate and harmful metals from process waters. These bioreactors are expected to be run for extended periods of time and may experience variations in the influent quality, such as increasing sulfate loading rate and decrease in pH, while being expected to function optimally. In this study we followed the sulfate removal rate and variation in microbial communities over a period of up to 333 days in three different up-flow anaerobic sludge blanket (UASB) bioreactors being submitted to increasing sulfate loading rate or decreasing pH. Sodium lactate was used as the sole carbon source and electron donor. All three bioreactors contained highly diverse microbial communities containing archaea, fungi and bacteria. *Sulfurospirillum* and *Desulfovibrio* were the most prominent bacterial genera detected in the bioreactors receiving the highest sulfate loading rates, and the greatest relative abundance of methanogenic archaea and the fungal genus *Cadophora* coincided with the highest sulfate reduction rates. In contrast, *Sulfuricurvum* was dominant in the bioreactor receiving influent with alternating pH, but its relative abundance receded in response to low pH of the influent. All bioreactors showed excellent sulfate removal even under extreme conditions in addition to unique responses in the microbial communities under changing operational conditions. This shows that a high diversity in the microbial consortia in the bioreactors could make the sulfate removal process less sensitive to changing operational conditions, such as variations in influent sulfate loading rate and pH.

**Supplementary Information:**

The online version contains supplementary material available at 10.1186/s13568-022-01438-2.

## Key points

- Influents with high sulfate loading rate or low pH were successfully treated.
- Microbial consortia showed versatility and adaption to changing operation.
- Unknown interactions among microbial groups makes interpretation challenging.

**Supplementary Information:**

The online version contains supplementary material available at 10.1186/s13568-022-01438-2.

## Introduction

Mining activities produce large quantities of effluents containing different hazardous compounds, such as sulfate and heavy metals, which can pose a threat to both the natural environment and fresh water sources (Heikkinen et al. 2005). Several methods, ranging from active to passive processes, exist in treating these effluents, including chemical precipitation, membrane treatment, ion-exchange and biological means (INAP 2003). Biological treatment methods are especially attractive both because of their cost-efficient ability to significantly reduce sulfate and heavy metals in waste waters and the possibility to recover valuable elements instead of disposing them to a waste sludge (Liamleam and Annachhatre 2007).

Sulfate reducing prokaryotes (SRP) are in the center of the biological treatment process. These microorganisms use sulfate as terminal electron acceptor when oxidizing organic carbon compounds, CO_2_ or H_2_ simultaneously producing sulfide (Muyzer and Stams 2008). Biological sulfate reduction (BSR) has been used in mining water treatment using different bioreactor configurations (for a review, see Kaksonen and Puhakka (2007)). These waters are characteristically rich in sulfate and have a low pH (Sánchez-Andrea et al. 2014), which can create challenges for a biological system. As sulfate is the terminal electron acceptor in BSR processes, increasing sulfate concentration or loading rate to a BSR bioreactor can have an inducing effect on sulfate reduction before the inhibiting levels are reached (Moosa et al. 2002; Al-Zuhair et al. 2008; Oyekola et al. 2010). In terms of pH, sulfate reducers generally prefer neutral conditions (Moosa and Harrison, 2006), but several studies have shown efficient sulfate removal even at very low pH with either naturally acidophilic or acclimatized populations (Lu et al. 2011; Ňancucheo and Johnson 2012, 2014).

In addition to sulfate reducers, a well-functioning BSR system may require complex interactions between different groups of microorganisms (Nagpal et al. 2000; Oyekola et al. 2010). Traditionally it is stated that with more complex organic substrates, such as waste materials, more assistance is needed from non-sulfate reducers to degrade these compounds to be suitable for sulfate reduction (Liamleam and Annachhatre 2007; Zhao et al. 2010). However, even with short chained organic compounds, such as ethanol or lactate, versatile microbial communities have been reported to reside in BSR bioreactors (Kaksonen et al. 2004; Bomberg et al. 2017), which may indicate both microbial co-operation as well as competition over the same substrates (Oyekola et al. 2010). Finding completely new microbial groups is not unusual either (Ňancucheo and Johnson 2012).

A broad range of studies in BSR have been published, but the relation between the process performance and changing conditions in the bioreactors to microbial communities inside the reactor in question has not been studied as extensively. This creates a gap in the understanding between the bioreactor operation and microbial consortia inhabiting the reactors (Hessler et al. 2020).

Our previously published works have focused on BSR in different operative conditions, by varying either sulfate loading rate (Salo et al. 2018) or influent pH (Salo et al. 2020). With a steady increase in sulfate loading rate, the BSR bioreactors continued to increase their sulfate removal rate, reaching a maximum of 15,000 mg/L*d (Salo et al. 2018), which is among the highest achieved in a BSR bioreactor (Liamleam and Annachhatre 2007; Qian et al. 2019). When testing the limits of acidity tolerance of a BSR bioreactor, the influent pH could be decreased down to 1.6 – 1.8 while maintaining an efficient sulfate removal rate (Salo et al. 2020). To have a functional BSR bioreactor with an influent pH as low as this is also quite exceptional (Elliott et al. 1998; Lu et al. 2011; Ňancucheo and Johnson 2014; Qian et al. 2019).

The current study focuses on the variety and changes in microbial populations during the operation of these BSR bioreactors and aims to discover clues for interdependence between the biological and chemical parameters, namely sulfate loading rate and influent pH. Microbial samples were analysed for their archaeal, fungal and bacterial consortia. In addition, the metabolic functions of the archaeal and bacterial communities were estimated using FAPROTAX (Additional files 1 and 2). The microbial communities were investigated in relation to time, sulfate loading rate and pH, and statistical analyses were conducted to find support for possible correlative relations.

## Materials and methods

### Bioreactors

In all experiments, the reactor vessel was a 0.7 L glass column operated as an up-flow anaerobic sludge blanket (UASB) reactor with the addition of 350 mL of carrier material (polymer or glass beads). Details of the bioreactor configurations and operations have been described in Salo et al. (2018, 2020). Altogether three different reactors were operated. Bioreactors BR 1 A and BR 1 B, described in Salo et al. (2018) (there named BR1 and BR2, respectively), were studied for the effect of sulfate loading rate using a pH neutral, 1600 mg SO_4_/L influent. Bioreactor BR 2, described in Salo et al. (2020), was stabilized with similar influent, but here the effect of a more acidic influent was examined. Inoculum for BR 1 A and BR 1 B was obtained from previous BSR bioreactor effluent and maintained in anoxic Postgate medium 63 (Postgate 1963; Leibniz-Institut DSMZ GmbH 2017). Similarly, the inoculum for BR 2 was obtained from BR 1 A and BR 1 B.

The influent for the experiments was prepared by agitating phosphogypsum (PG) waste material either with only ion-exchanged water (Salo et al. 2018) or with increasing sulfuric acid concentration (Salo et al. 2020). Sodium lactate was used as the carbon source and electron donor for all experiments, and its dose was altered depending on the sulfate concentration of the PG leachate, assuming stoichiometric reaction (Salo et al. 2020). Solution containing sodium lactate and other nutrients was kept refrigerated and fed to the bioreactors together with PG leachate, as described in Salo et al. (2018). Sample collection of reactor effluent for chemical and microbial analyses was conducted from a sampling tube near the top of the reactor vessel, providing a representative liquid sample from the bioreactor solution. Microbial samples (1.5 mL) were collected to Eppendorf tubes and kept frozen (below − 20 °C) after sampling.

### Increasing sulfate loading rate

After reaching a steady operation with BR 1 A and BR 1 B (Salo et al. 2018), the sulfate loading rate to the bioreactors was increased steadily from 1800 to 8000 mg/L*d over a period of 142 days. After a stable operation phase of 95 days, a stress test was conducted where the sulfate loading rate was increased from 8000 to 23,500 mg/L*d over a period of 21 days.

### Increasing influent acidity

The operation of BR 2 (Salo et al. 2020) was first stabilized with an influent of average pH 6.0 and containing up to 1800 mg/L of sulfate. Then 95% H_2_SO_4_ was used to decrease the influent pH first to approximately 1.8 and further to 1.3, with sulfate concentrations increasing to 2800 and 3800 mg/L, respectively, before feeding to the bioreactor. This gradual increase in influent acidity was conducted twice during the operation of the bioreactor.

### Analytical methods

Measurements from reactor influents and effluents included pH and redox potential observation with a Consort multi-parameter analyzer C3040 (Turnhout, Belgium) with Van London-pHoenix Co. electrodes (Ag/AgCl in 3M KCl, Houston, Texas, USA). Sulfate measurements were conducted with the Hach Lange kit (LCK 353) and analysed with a Hach Lange spectrophotometer (DR 3900, Manchester, UK). Acetate was measured according to standard SFS-EN ISO 10304-1:2009 by Metropolilab Oy, Helsinki.

The DNA was extracted from the collected samples using the NucleoSpin Soil DNA extraction kit (Macherey–Nagel, Düren, Germany). The samples were first thawed on ice where after the biomass was pelleted by centrifugation using Eppendorf 5417R table-top centrifuge (Eppendorf, Hamburg, Germany) at 20,800*g* for 10 min. The supernatant was removed leaving 100 µL in which the pellet was dissolved. The dissolved pellets were transferred to the bead tubes provided in the DNA extraction kit. SL1 lysis buffer and Enhancer solution SX were added to the bead tubes according to the manufacturer’s instructions. The microbial cells were lysed by placing the bead tubes vertically on a Vortex Genie 2.0 shaker and vortexed for 5 min after which the DNA extraction proceeded according to the manufacturer’s instructions. The DNA was eluted in 100 µL elution buffer EB. The DNA content of the samples was checked using the Qubit 2.0 (Invitrogen) spectrophotometer with the dsDNA HS Assay kit (Invitrogen).

Amplicons for characterizing the microbial consortia in the bioreactors were obtained by PCR. Bacterial and archaeal 16S rRNA genes were targeted with primers Bact_341F/ Bact_805R (Herlemann et al. 2011) and S-D-Arch-0349-a-S-17/S-D-Arch-0787-a-A-20 (Klindworth et al. 2013), respectively, whereas fungal ITS1 regions were targeted using primers ITS1 and ITS2 (White et al. 1990; Gardes and Bruns 1993). The forward primers were equipped with 9-nucleotides long barcodes unique for each sample and both forward and reverse primers contained adapter sequences for the Iontorrent platform at their 5′ ends. Parallel 25-μL reactions were prepared for all samples in order to reduce PCR bias. The PCR mix consisted of 1 × MyTaq™ Red Mix (Bioline, London, UK), 20 pmol of each primer, up to 25 μL molecular-biology-grade water (Sigma) and 2 μL of template DNA. The PCR reactions were run on an Eppendorf MasterCycler gradient thermocycler (Eppendorf, Hamburg, Germany). The amplification program consisted of an initial denaturation step at 95 °C for 3 min, followed by 40 cycles of 15 s at 95 °C, 15 s at 50 °C and 15 s at 72 °C, with a final elongation of 30 s at 72 °C. Correct sizes of the amplicons were verified with agarose gel electrophoresis, whereafter the parallel amplicons were combined and sequenced in one direction on the Ion Torrent PGM sequencing on a 316 chip at Bioser Oy (Oulu, Finland), where the amplicons were purified and size checked before sequencing.

The sequence data was analysed using the mothur software package version 1.43.0 (Schloss et al. 2009). The Silva reference database version 138 for bacterial and archaeal 16S rRNA genes (Pruesse et al. 2007; Quast et al. 2013) for and the UNITE database version 8 for fungal ITS sequences (Kõljalg et al. 2013; UNITE Community 2017; Nilsson et al. 2019) were used as reference databases for sequence identification. The mothur analysis pipeline consisted of an initial sequence quality check, where adapter and barcode sequences were removed. The sequences were trimmed to a minimum length of 200 bp, allowing one nucleotide difference in the primer sequence and no mismatches in the barcode sequences, maximum 8 nucleotide long homopolymer stretches, no ambiguous nucleotides, and a quality average of 20 over 40 nucleotide window size. The sequence data was dereplicated using unique.seqs after which the bacterial and archaeal sequence reads were aligned (align.seqs) against the Silva 138 full database, which had been optimized to include only the region targeted by the bacterial and archaeal primers. The alignment was subsequently screened (screen.seqs) to include only sequences covering the targeted area of the alignment (latest starting position 6430 and 6452, earliest ending position 14,000 and 15,000 for bacteria and archaea, respectively). Gaps spanning the whole screened alignment were removed and the ends of the alignments were cut (filter.seqs). Redundant sequences and possible sequencing errors were removed by running the unique.seqs followed by the pre.cluster command. Chimeric sequences were identified (chimera.vsearch) and removed from the data. The sequence reads were classified using the Silva 138 database after which all non-bacterial and non-archaeal sequences were removed from the bacterial and archaeal sequence data, respectively. A distance matrix was built on the aligned sequences using dist.seqs, and the sequences were clustered into Operational Taxonomuc Units (OTUs) sharing 97% sequence homology using the cluster command. A table of the abundance of each OTU in each sample was produced (make.shared) and the OTUs were classified based on the sequence read classification.

The fungal ITS sequence data was trimmed using the same quality parameters as for the bacterial sequence data, dereplicated using unique.seqs, but not aligned. Chimeric sequences were identified with chimera.vsearch and removed. Sequences were classified (classify.seqs) against the Unite version 8 full ITS database. All non-fungal sequences were removed. The unaligned sequence reads were clustered into OTUs with 97% sequence homology and processed as described for bacteria and archaea. OTUs present in the DNA-extraction reagent controls and PCR reagent controls were evaluated and removed manually from the data.

PAST software version 4.03 (Hammer et al. 2001) was used in the statistical analyses of the data. These analyses included tests for normality, canonical correspondence and Tukey’s pairwise method. The analyses were conducted for all bioreactors together as well as separately for BR 1 A and B and BR 2 bioreactors. In addition, Chao1 and Shannon indices were determined from the OTUs of each microbial domain. Metabolic functions for archaea and bacteria were investigated using FAPROTAX (Functional Annotation of Prokaryotic Taxa) version 1.1 (Additional files 1 and 2; Louca et al. 2016), using a modified database (Bomberg 2020).

## Results

### Bioreactor performances

BR 1 A and BR 1 B were operated in parallel (Salo et al. 2018) and both bioreactors began efficient sulfate reduction soon after the continuous operation was started on day 13 (Table 1). During days 52–57, the bioreactors received an overdose of nutrients (ten times the calculated amount of nutrients and substrate), and sulfate reduction temporarily halted. Both bioreactors had recovered by day 135, but an extra 100 mL inoculum dose was introduced to BR 1 A on day 98 to ensure full recovery. A slow and steady increase in sulfate loading rate from 1800 to 8000 mg/L*d was introduced to both bioreactors between days 71 and 213, followed by a stable loading phase from day 213 to 308. From day 311 until day 333, a stress test was conducted, where the sulfate loading rate was increased more frequently and strongly from 8000 to 23,500 mg/L*d. During the experiments, both BR 1 A and BR 1 B continued to increase their sulfate reduction rate even during the final stress test, when the reduction rates stabilized to approximately 15,000 mg/L*d. The redox potentials were generally low during the operation (− 299 to − 370 mV), indicating an anaerobic environment suitable for BSR. Only when the nutrient overdose occurred, the redox potentials slightly increased in both bioreactors, reaching the highest level of -129 mV on day 92 in BR 1 A before the addition of extra inoculum. The concentration of acetate showed a sudden leap to up to 16,000 mg/L during the nutrient overdose phase, stabilized afterwards to approximately 300–500 mg/L, and then nearly doubled towards the end of the stress test.

**Table 1:** Operative details of the BSR bioreactors regarding pH and sulfate. Microbial samples were analysed on the days listed in the table for each bioreactor. HRT = hydraulic retention time in the carrier material

		Influent				Effluent				
Bioreactor	Day	pH	SO_4_ (mg/L)	SO_4_ load (mg/L*d)	HRT (h)	pH	Redox (mV)	SO_4_ (mg/L)	SO_4_ reduction (mg/L*d)	Acetate (mg/L)
BR 1 A	49	5.9	1600	1600	23.6	7.2	− 357	290	1300	5
	57	5.2	1600	1800	21.2	6.2	− 290	830	700	16,000
	92	5.4	1600	2300	17.0	7.0	− 129	900	800	< 2
	135	5.8	1600	4700	8.2	7.2	− 299	140	3500	54
	251	5.6	1600	8000	4.8	7.3	− 345	410	6000	350
	315	5.5	1600	13,700	2.8	7.0	− 346	460	9900	490
	319	5.7	1600	16,500	2.3	6.9	− 333	120	14,200	n.a
	322	5.8	1600	19,300	2.0	6.9	− 336	520	13,200	565
	333	5.6	1600	23,500	1.6	6.9	− 333	600	15,000	850
BR 1 B	49	5.9	1600	1600	23.6	7.0	− 370	310	1300	9
	57	5.2	1600	1800	21.2	6.2	− 251	920	590	15,000
	92	5.4	1600	2300	17.0	7.0	− 301	330	1400	437
	135	5.8	1600	4700	8.2	6.8	− 304	510	2600	450
	251	5.6	1600	8000	4.8	7.5	− 332	440	5800	560
	315	5.5	1600	13,700	2.8	6.7	− 328	460	9900	690
	322	5.7	1600	19,300	2.0	6.7	− 329	480	13,700	660
	329	5.8	1600	22,800	1.7	6.9	− 336	630	14,100	1100
	333	5.6	1600	23,500	1.6	6.7	− 335	560	15,500	960
BR 2	52	6.3	1700	1600	24.6	7.1	− 380	570	1000	83
	70	6.1	1700	2100	18.7	7.4	− 353	250	1800	140
	95	6.1	1700	2500	16.0	7.4	− 416	430	1800	280
	110	1.8	2900	1800	37.6	6.8	− 353	320	1600	n.a
	115	1.8	2900	2600	25.2	6.5	− 364	540	2100	1300
	124	1.3	3900	2400	37.6	5.8	− 276	1400	1500	2300
	138	5.9	1900	1700	25.2	7.3	− 346	690	1200	380
	166	5.8	1900	2100	20.5	7.6	− 390	380	1700	44
	174	1.7	2700	1600	37.6	7.2	− 354	120	1600	480
	194	1.6	2700	2500	25.2	6.8	− 290	470	2000	670
	199	1.3	3400	2100	37.6	6.1	− 269	1200	1300	1600
	208	1.3	3400	2100	37.6	5.3	− 176	2000	800	2400

n.a.: not analyzed

The operation of BR 2 was started similarly to BR 1 A and BR 1 B, and a steady sulfate reduction was reached during the first 95 days using a near neutral pH influent (Salo et al. 2020) (Table 1). Afterwards, the acidity tolerance of BR 2 was tested twice with influent pH values 1.8 and 1.3 during days 95–124 and days 166–208. In these experiments, BR 2 could withstand the pH 1.8 influent and even slightly increase the sulfate reduction rate, but pH 1.3 influent caused the bioreactor performance to rapidly decline each time. Redox potentials remained low during most of the operation, however, increasing acidity also caused the redox potentials to increase. Acetate concentration strongly increased as the influent pH decreased.

### Microbial consortia

Due to the need to maintain anaerobic conditions in the bioreactors, they were not opened for sampling and thus these results focus on the microorganisms in the effluent from the bioreactors. Most of the samples from the bioreactors contained a low number of archaeal sequences (Additional file 2: Table S1) and therefore the results concerning archaeal consortia are not shown. In BR 1 A, most archaea were found from day 251 to 333, and these consisted mostly of the archaeal genera *Methanospirillum* (5–87%), *Methanosarcina* (2–91%) and *Methanomethylovorans* (4–11%), co-occurring with the highest sulfate loading rate. In BR 1 B, samples only on days 315 and 322 could be deemed representative, and then the archaeal population consisted also mainly of *Methanospirillum* (44–46%), *Methanosarcina* (7–9%) and *Methanomethylovorans* (44–45%). According to one sampling on day 166 in BR 2, the archaeal population was dominated by *Methanospirillum* (86%) and *Methanomethylovorans* (3%). The metabolic predictions with FAPROTAX (Additional file 1) indicated that methanogenesis utilizing different carbon substrates was the most common archaeal metabolism in the bioreactors.

**Fig. 1:**
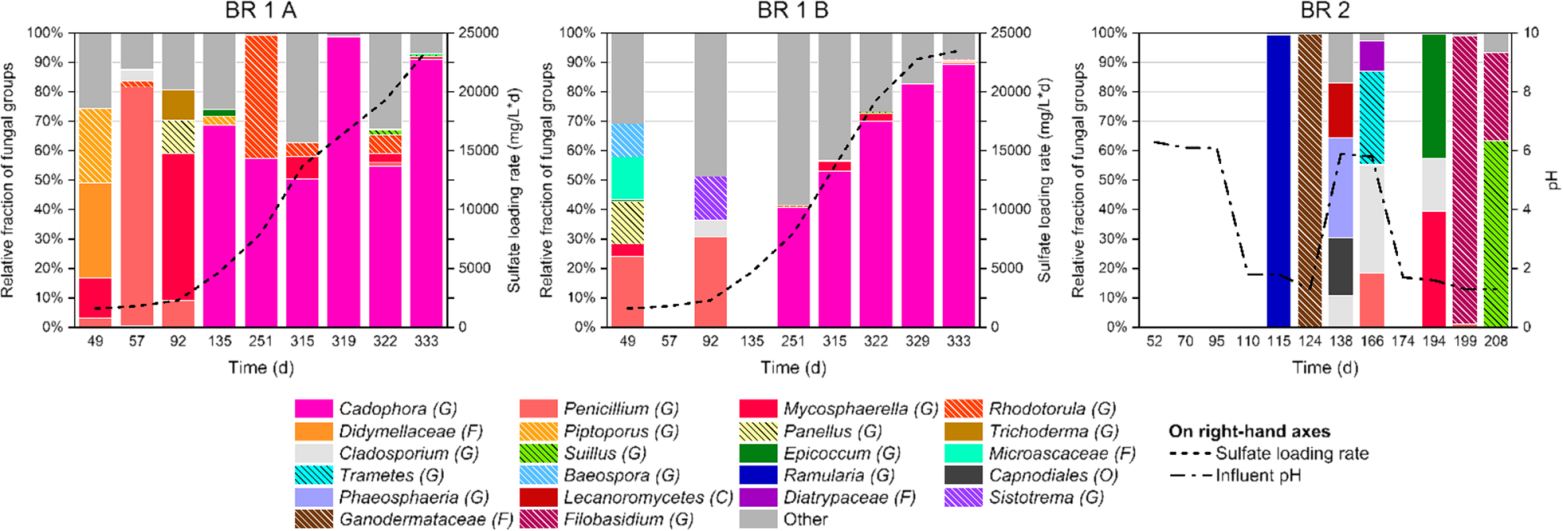
Fungal groups found in BSR bioreactor effluents during the operation and the main operational parameters. Samples on days 57 and 135 in BR 1 B and 52, 70, 95, 110 and 124 in BR 2 were excluded because of low sequence counts. All groups could not be identified to genus level. C: class; O: order; F: family; G: genus; other: unknown fungi and fungal groups with daily fractions below 10%. Fungal groups belonging to the phylum Basidiomycota are marked with line patterns while unmarked groups belong to the phylum Ascomycota

Most fungal groups in BR 1 A and BR 1 B belonged to the Ascomycota phylum while in BR 2 both Basidiomycota and Ascomycota were nearly equally represented (Fig. 1). The *Cadophora* genus became dominant in BR 1 A and BR 1 B (40–99%) after reactor performance had stabilized after day 135. In the beginning, when the nutrient overdose occurred between days 52 and 57, other genera such as *Penicillium* dominated in both bioreactors (3–81%), with fractions of *Mycosphaerella* (up to 50%), *Piptoporus* (up to 25%) and unclassified genera of the family *Didymellaceae* (up to 32%) in BR 1 A, and *Panellus* and unclassified genera of the family *Microascaceae* (both up to 15%) in BR 1 B. Fungal groups in these bioreactors reacted differently to increasing sulfate loading rate, as in BR 1 A the fraction of *Cadophora* fluctuated more inconsistently than in BR 1 B, where *Cadophora* increased steadily from 41% of stabilized operation to 89% of the highest sulfate loading rate. The other major known fungal genus, *Rhodotorula*, was only apparent during stable operation on day 251 with 42% and was not significantly present during the stress test. In BR 2, the fungal groups were much more incoherent compared to the other two bioreactors and several sampling days were excluded due to a low number of sequences obtained from the samples. No connection between the fungal groups in BR 2 and the bioreactor performance was observed.

**Fig. 2:**
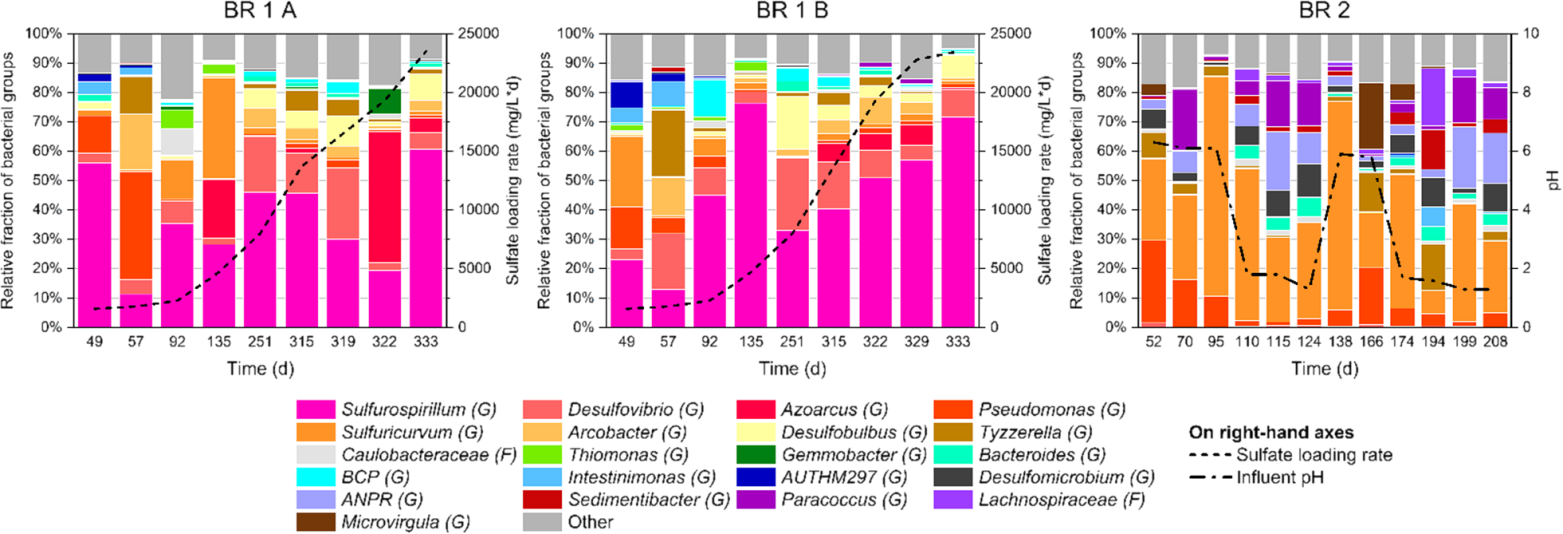
Bacterial groups found in BSR bioreactor effluents during the operation and the main operational parameters. All groups could not be identified to genus level. F: family; G: genus, *BCP Burkholderia-Caballeronia-Paraburkholderia*, *ANPR Allorhizobium-Neorhizobium-Pararhizobium-Rhizobium*, Other: unknown bacteria and bacterial groups with daily fractions below 6%

The bacterial consortia showed more variation during the different phases of operation and differences between the bioreactors were notable (Fig. 2). In BR 1 A and BR 1 B, the most distinctive genus was *Sulfurospirillum* (11–76%), followed by *Desulfovibrio* (3–25%) and *Azoarcus* (up to 20%). *Pseudomonas* was present in the beginning (5–37%), but greatly diminished after the nutrient overdose (0.4–4%). BR 2 differs from the other two bioreactors, as here *Pseudomonas* is more abundantly present throughout the operation (1–28%), although competed with the most abundant genus *Sulfuricurvum* (8–75%) and followed by *Allorhizobium-Neorhizobium-Pararhizobium-Rhizobium* (0.5–21%), *Paracoccus* (0.3–21%) and *Desulfomicrobium* (1–11%).

Similar to the fungal consortia, the bacteria in BR 1 B behaved more consistently than in BR 1 A when sulfate loading rate was increased (Fig. 2). *Sulfurospirillum*, responsible for the respiration of both sulfur and nitrogen compounds according to the metabolic predictions (Additional file 1 and Additional file 2: Fig. S1), was the dominant group in nearly all samples after the start-up in both bioreactors, with more fluctuation in BR 1 A than in BR 1 B. The fraction of sulfate-reducing *Desulfovibrio* decreased from 19–25% to 6–9% as sulfate loading rate increased, while another sulfate reducer *Desulfobulbus* remained relatively stable (1–10%) after day 251.

**Fig. 3:**
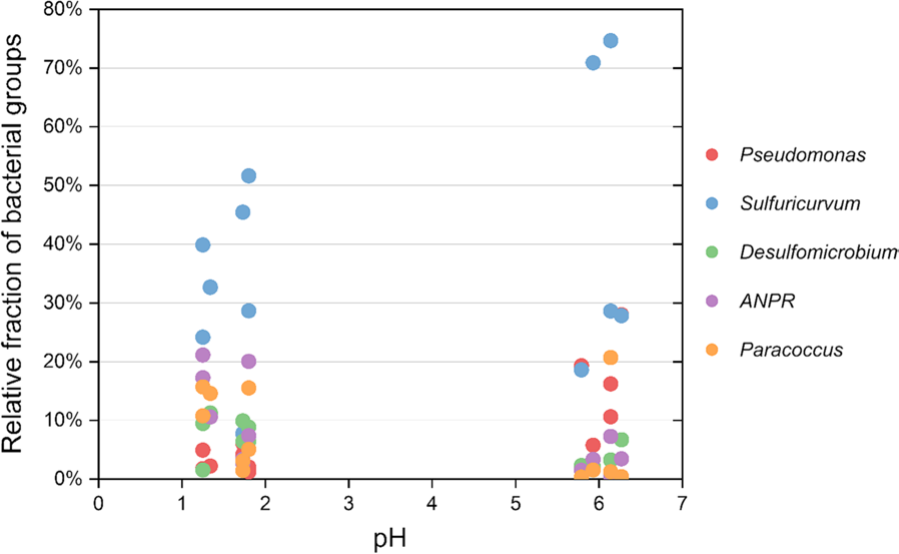
Main bacterial genera and influent pH in BR 2. *ANPR*:  *Allorhizobium-Neorhizobium-Pararhizobium-Rhizobium*

In the case of the most prominent bacterial genera in BR 2 (Fig. 3), *Sulfuricurvum* was dominating both with lower and higher influent pH, although the fractions decreased from 75 to 24% with more acidic influent. *Pseudomonas* also decreased from mostly above 10% of the bacterial consortium to below 6% together with decreasing pH. *Allorhizobium-Neorhizobium-Pararhizobium-Rhizobium* increased from less than 10% to above 20% with decreasing pH, and similar behavior was observed from *Paracoccus* (mostly from less than 5% to above 10%).

### Statistical analyses

Normality tests for all bioreactors in terms of the domains Fungi and Bacteria all followed the normal distribution of both Shapiro–Wilk and Anderson–Darling with p-values < 0.05 (10,000 calculations). However, occasionally the separate bioreactor analyses did not pass these normality tests in terms of all microbial groups or chemical parameters. This is probably due to the low amount of sample points when the bioreactors were analysed separately.

**Fig. 4:**
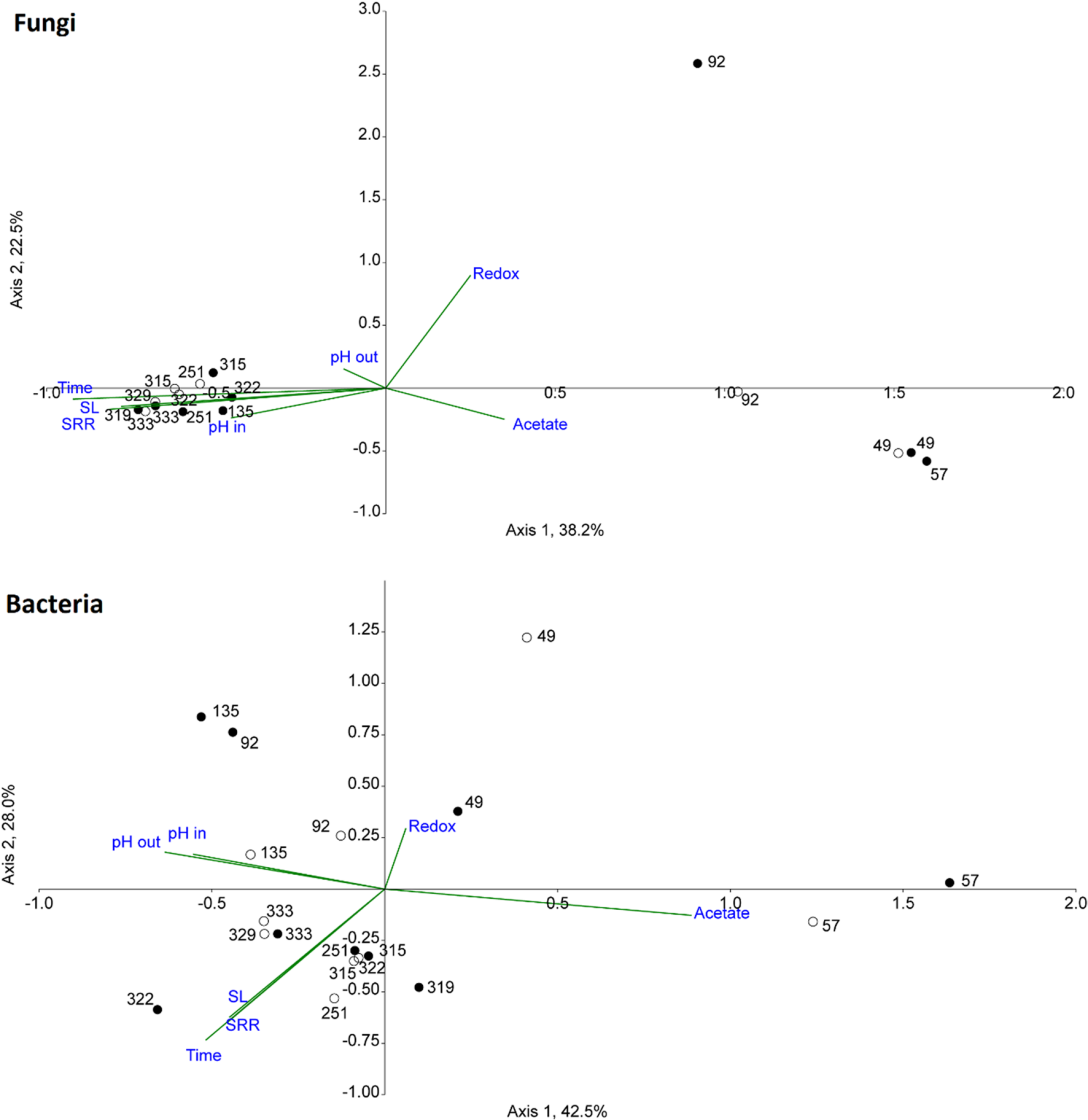
CCA analyses of BR 1 A and BR 1 B microbial data against the measured parameters, axes show percentage of variance. Data point labels refer to sampling dates. Filled spheres: BR 1 A, open spheres: BR 1 B, SL:  sulfate loading rate, SRR: sulfate removal rate

The Canonical correspondence analysis (CCA) showed strong dependency between sulfate loading/reduction rate and BR 1 A and BR 1 B microbial data over other measured parameters (Fig. 4). This correspondence was especially clear during the final days of operation when the stress test was conducted (days 315–333). Particularly the fungal consortia followed closely both sulfate loading and reduction rate, which highlights the possible importance of fungal groups for bioreactor operation. According to the CCA of BR 2 microbial data (Additional file 2: Fig. S3), only the fungal consortia indicated correspondence with influent pH, however, the majority of data points were excluded from the analysis due to low sequence counts (Fig. 1). Bacterial consortia in BR 2 showed dependence to acetate concentration (Additional file 2: Fig. S3), which can be thought to be an indirect indication of pH dependency, as the acetate concentration increased as a result of decreasing influent pH (Table 1).

Tukey’s pairwise method also showed strong correlation between sulfate loading rate and microbial groups (p < 0.05), whereas with influent pH the correlation was not detected, probably because of only few data points.

According to Chao1 and Shannon indices, the richness and diversity of the microbial populations in BR 1 A and BR 1 B were slightly above those of BR 2 (Additional file 2: Tables S2, S3). When sulfate loading rate was increased in BR 1 A and BR 1 B, the fungal community richness slightly increased only in BR 1 A and remained approximately the same in BR 1B, however Shannon’s diversity index for the fungal consortia decreased in both bioreactors as the sulfate loading rate was increased. No clear changes in the richness and diversity index of the bacterial consortia were detected. In BR 2 (Additional file 2: Tables S2, S3), Shannon’s diversity index of the fungal consortia decreased together with decreasing influent pH, whereas the richness remained stable despite changes in pH. Both the richness and diversity of bacteria increased in BR 2 as the pH decreased, except on day 208 when the bioreactor was nearing failure and both parameters decreased sharply.

## Discussion

### Effect of sulfate loading rate

The effect of changing sulfate loading rate on archaea, fungi and bacteria was examined in bioreactor BR 1 A and BR 1 B effluents (Figs. 1, 2). According to many studies, archaea can be difficult to detect in BSR bioreactors (Ňancucheo and Johnson 2014; Santos and Johnson 2017). However, when archaeal populations have been reported, they are often methanogens (Rezadehbashi and Baldwin 2018), which is in agreement with our study. In nature, methanogens and sulfate-reducing bacteria compete over certain substrates (e.g. hydrogen and acetate), and if sulfate is also present, the sulfate reducers will be victorious, although co-existence of these microbial groups is possible (Oremland and Polcin 1982; Sela-Adler et al. 2017). Archaeal groups from the same orders (*Methanomicrobiales* and *Methanosarcinales*) were also found by Baldwin et al. (2015).

Fungi are rarely addressed in BSR studies, although their presence has been noted and their likely influence on the performance of a bioreactor can be great together with archaeal and bacterial groups (Bomberg et al. 2017). For this reason, it is difficult to assess the present findings and question their relation towards the bioreactor operation (Fig. 1). Nevertheless, the relative abundance of *Cadophora* stabilized before the stress test in both bioreactors and was not disturbed by the increasing sulfate loading rate. In contrast, it prospered while other fungal groups diminished, indicating high tolerance to changing environmental conditions for this fungal genus. Aldossari and Ishii (2021) detected *Cadophora* in soil and woodchip bioreactors at relatively low temperatures (5–15 °C) and confirmed the nitrate-reducing capability of this genus. It is likely that *Cadophora* is an important contributor in the nitrogen cycle also in our BSR bioreactors, especially when the sulfate loading was gradually increased. In general, fungi can also take part in symbiotic substrate utilization together with bacteria. Drake and Ivarsson (2018) suggested that in deep subsurface ecosystems, where hydrogen gas is thought to be the main substrate for sulfate-reducing bacteria, fungi may play a significant role in hydrogen production. It is clear that more research is desperately needed to understand the correlations between BSR bioreactors and fungi.

It has been noted in several studies (e.g. Bomberg et al. 2017; Salo 2017; Rezadehbashi and Baldwin 2018; Huang et al. 2020) that the fraction of SRB in relation to other bacteria can be surprisingly low even though sulfate removal is efficient. In BR 1 A and BR 1 B (Fig. 2), the fraction of sulfate reducers decreased even though the sulfate removal rate increased during the stress test, which can mean that either the remaining sulfate reducers were acclimated to utilize sulfate more rapidly or other unknown groups had either directly or indirectly participated in sulfate removal. The same sulfate reducers were found from ethanol-fed bioreactors (Bomberg et al. 2017) as from bioreactor utilizing more complex organic waste materials (Baldwin et al. 2015), including *Desulfovibrio*, *Desulfobulbus* and *Desulfomicrobium*. *Desulfovibrio* and *Sulfurospirillum* were also found by Giordani et al. (2019) in an operational phase with high sulfate loading rate and no supplied nutrients, although larger bacterial groups were represented by *Syntrophobacter*, *Longilinea* and *Geobacter*, which were non-existent in our study. Interestingly, Hessler et al. (2020) found that *Desulfovibrio* decreased as they increased sulfate loading rate, whereas in our study the relative abundance of this genus first increased through the operation during slow increase in sulfate loading rate, and then slightly diminished during the stress test. Completely different SRB genera were also found by Icgen and Harrison (2006), who studied the effect of high sulfate concentration on a mixed bacterial culture, and identified groups such as *Desulfonema*, *Desulfobacterium*, *Desulfobacter* and *Desulfococcus*. This highlights the difficulty of direct comparison between studies, as mixed cultures are always unique and ever changing, and different microbial identification methods can yield varying results.

The great variability in acetate concentrations during the operation of BR 1 A and BR 1 B (Table 1) may have been caused by several reasons. During the start-up phase, the exceptionally low amount of acetate could have resulted either from the presence of complete oxidizers, which are able to degrade the substrate completely to carbon dioxide, or the contribution of other microorganisms consuming the formed acetate (Muyzer and Stams 2008). The increasing concentrations of acetate in the effluents could also indicate a shift from sulfate reduction to substrate fermentation inside the bioreactors (Dar et al. 2008). The failure of the bioreactors during the nutrient overdose phase might have occurred because of acetate inhibition (Baronofsky et al. 1984) caused by the inability or lack of SRP or other microorganisms able to perform the complete oxidation of lactate. The increasing acetate concentration together with increasing sulfate loading rate during the stress test could again possibly indicate a beginning inhibition.

In terms of statistics, Oyekola et al. (2007) noted an increase in microbial diversity when sulfate loading rate was increased. They postulated that more diverse communities can better tolerate changing conditions and improve process performance. In our study, however, even though Chao1 richness index and Shannon’s diversity index of the bacterial consortia had no clear correlation to sulfate loading rate in either BR 1 A or BR 1 B (Additional file 2: Table S3), the sulfate removal continued to increase until the end of the experiment. It is possible that the richness and diversity of the microbial communities in BR 1 A and BR 1 B had stabilized to a near optimal, where different groups had found their niche in the system during long operation and not even substantially increasing sulfate loading rate could overthrow this balance.

### Effect of acidity

The effect of varying influent pH could only be reasonably estimated on the bacterial domain in BR 2 effluent (Figs. 1, 2). Species in the *Sulfuricurvum* genus, the dominant bacterial group in BR 2, are responsible for oxidation of sulfur compounds and denitrification, according to the metabolic predictions (Additional file 1 and Additional file 2: Fig. S2). This would mean that *Sulfuricurvum* could have consumed sulfide and produce either elemental sulfur or sulfate, potentially decreasing the overall sulfate reduction rate in BR 2 (Table 1, Fig. 2). *Pseudomonas* genus is in charge of respiration and degradation of several compounds, including sulfur and nitrogen compounds (Additional file 1). *Pseudomomas* cannot withstand low pH (Brenner et al. 2005a), so its fraction was expectedly lower with more acidic influents (Fig. 3). *Allorhizobium-Neorhizobium-Pararhizobium-Rhizobium* is listed under nitrogen fixation in the metabolic predictions (Additional file 1) and at least some species of this genus are aerobic (Brenner et al. 2005b). Some species can also be acid-tolerant (Gopalakrishnan et al. 2015), which may explain the increase of fraction with lowering pH, possibly due to less competition with more pH sensitive groups (Fig. 3). Species under the *Paracoccus* genus are known for oxidation of sulfur and nitrogen compounds (Additional file 1) in aerobic environment (Brenner et al. 2005b). Their increase in relative abundance during acidic conditions may have contributed to lower sulfate reduction rate, as more sulfide was oxidized back to sulfate (Fig. 3). Even though the conditions in BR 2 were anaerobic throughout the operation according to the measured redox potentials (Table 1), it is still not unusual to discover aerobic microorganisms alongside anaerobic during a long experiment (Salo 2017). As the acetate concentration increased simultaneously with decreasing influent pH (Table 1), a similar substrate oxidation disturbance as with increasing sulfate loading rate in bioreactors BR 1 A and BR 1 B is indicated.

As mine waters containing sulfate can be extremely acidic, the effect of low pH on sulfate-reducing microorganisms is a widely studied topic. Zhao et al. (2017) operated a bioreactor with gradually decreasing influent pH from 4.5 to 2.5. During the lowest influent pH, *Desulfobacter* and *Desulfovibrio* were the dominant sulfate reducers, whereas in our study *Desulfomicrobium* was practically the only sulfate reducer during the experiment. The fraction of *Desulfomicrobium* remained approximately 10% when influent pH was 1.6–1.8. Ňancucheo and Johnson (2014) researched a sulfidogenic bioreactor, which was maintained at low pH (2.8–4.5) while treating highly acidic (pH 1.3–3.0) influents. They had excellent sulfate removal results at low pH, similar to our bioreactor operated with pH 1.6–1.8 influent. However, *Desulfosporosinus* was the main genus responsible for sulfate reduction in Ňancucheo and Johnson’s (2014) low pH environment. This same finding was confirmed in other studies as well (Ňancucheo and Johnson 2012; Santos and Johnson 2017; González et al. 2019).

Zhao et al. (2017) reported a decrease in microbial richness as the influent acidity was increased, and this was also supported by other studies (e.g. Montoya et al. 2013), while our findings were the opposite in terms of bacterial communities (Additional file 2: Table S3). Our results contradict the general assumption that the biodiversity of microorganisms is lower in acidic environments. Here the reason could be that richer communities can better adapt to changes in pH, as was suggested by Oyekola et al. (2007) in terms of sulfate loading rate.

During the operation of BR 2, the sulfate concentration increased slightly because the influent pH was adjusted with H_2_SO_4_, which could have influenced the microbial consortia. However, this was assumed to be mostly overpowered by the effect of highly acidic pH. As the effect of sole sulfate concentration could not be verified from these experiments, the debate on this matter is omitted from this work.

In conclusion, the sulfate-reducing bioreactors of this study showed increasing sulfate removal rate while sulfate loading rate was gradually increased. Influent acidity was well tolerated down to pH 1.6–1.8. All bioreactors exhibited diverse microbial communities, which could support sulfate removal even during extreme process conditions. *Sulfurospirillum* and *Sulfuricurvum* were the most abundant bacterial genera in the bioreactors, and sulfate reducers were also well represented, the dominant genera being *Desulfovibrio*, *Pseudomonas*, *Desulfobulbus* and *Desulfomicrobium*. Bacterial richness and diversity remained relatively stable through increasing sulfate loading rate and increased together with influent acidity. The low amount of representative archaeal samples and the scarcity of fungal research in sulfate-reducing environment hindered the interpretation of these domains and their connection to bioreactor operation.

Experimental issues, such as challenges to recover enough microbial DNA from small volume bioreactors for proper analyses, as was also reported by Santos and Johnson (2017). In our reactor configuration, it would be very difficult to obtain several sludge samples for microbial analyses without disturbing the system and affecting both the current and future operation of the bioreactor. By taking effluent samples from the bioreactor, we acknowledge that some microbial information might be left undiscovered. As reported by Hessler et al. (2020), microbial communities in the free-floating phase can greatly differ from those attached or associated to a biofilm. However, continuous sampling offers the opportunity to observe long-term bioreactor operation and correlations between biological and chemical parameters, while keeping the bioreactor mostly undisturbed. Different reactor configurations and larger operating volumes could offer more flexibility in sampling and supply more accurate information on microbial communities.

## Supplementary Information

**Additional file 1.** Archaeal and bacterial metabolic functions in bioreactors according to Functional Annotation of Prokaryotic Taxa (FAPROTAX) (Louca et al. 2016) with a modified database (Bomberg 2020).

**Additional file 2.** Additional figures and tables.

## Data Availability

All data generated or analysed during this study are included in this published article and its Additional files 1 and 2. The sequence data is available from the European Nucleotide Archive (www.ebi.ac.uk/ena/) under Study Number PRJEB50652.
