# Supplementary material for: Sulfate-reducing bioreactors subjected to high sulfate loading rate or acidity: variations in microbial consortia
Source: AMB Express. 2022 Jul 16;12:95. doi: 10.1186/s13568-022-01438-2 (PMC9288570; doi:10.1186/s13568-022-01438-2)
Supplement: Supplementary file 2 — Additional file 2. Additional figures and tables. [file 13568_2022_1438_MOESM2_ESM.pdf]

AMB Express

Sulfate-reducing bioreactors subjected to high sulfate loading rate or acidity: Variations in microbial consortia

Marja Salo, Malin Bomberg

VTT Technical Research Centre of Finland Ltd, P.O.Box 1000, 02044 VTT, Finland

marja.salo@vtt.fi, +358406428222

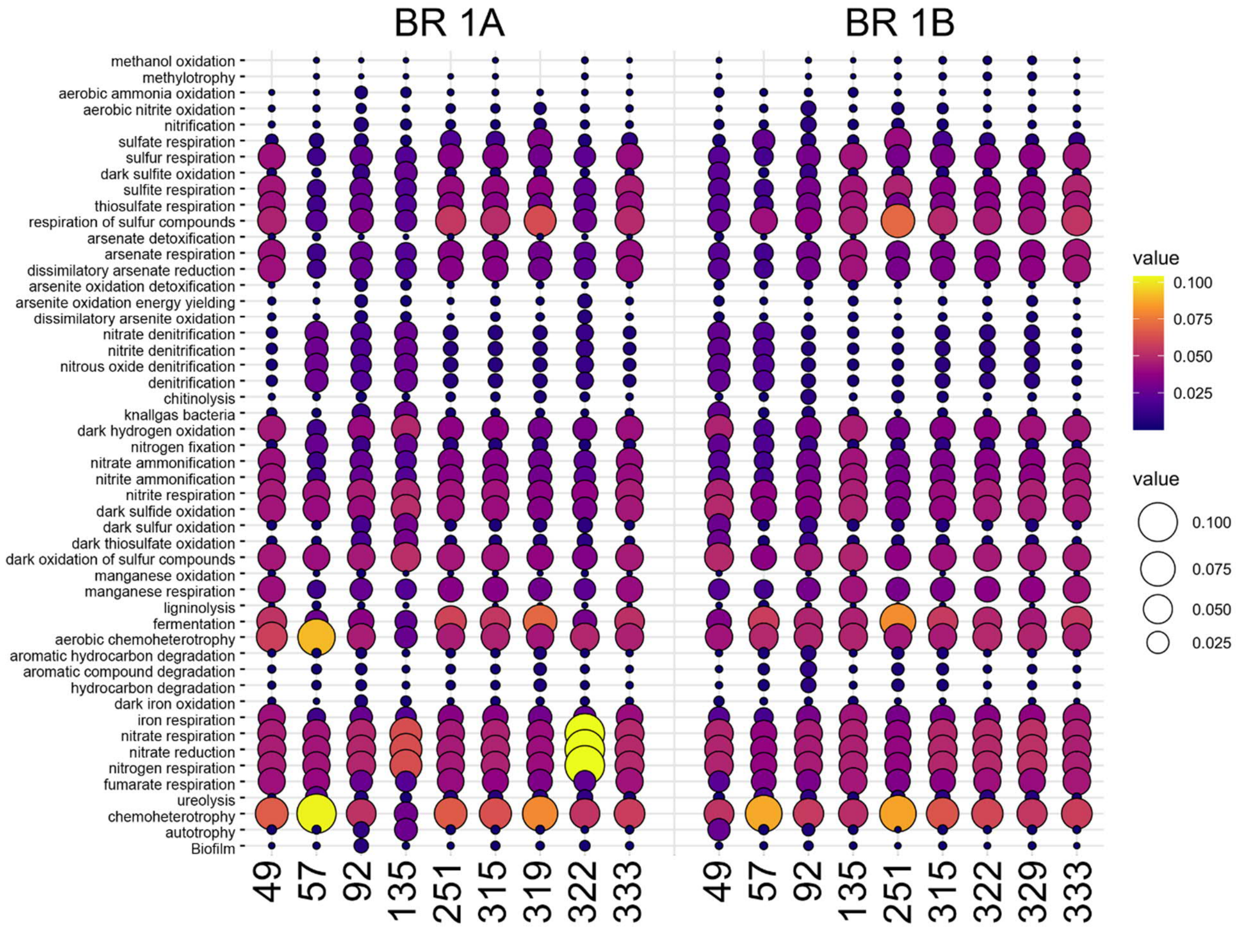

Fig. S1 Metabolic predictions of bacteria in BR 1 A and BR 1 B by sampling day according to FAPROTAX (Louca et al., 2016; Bomberg, 2020).

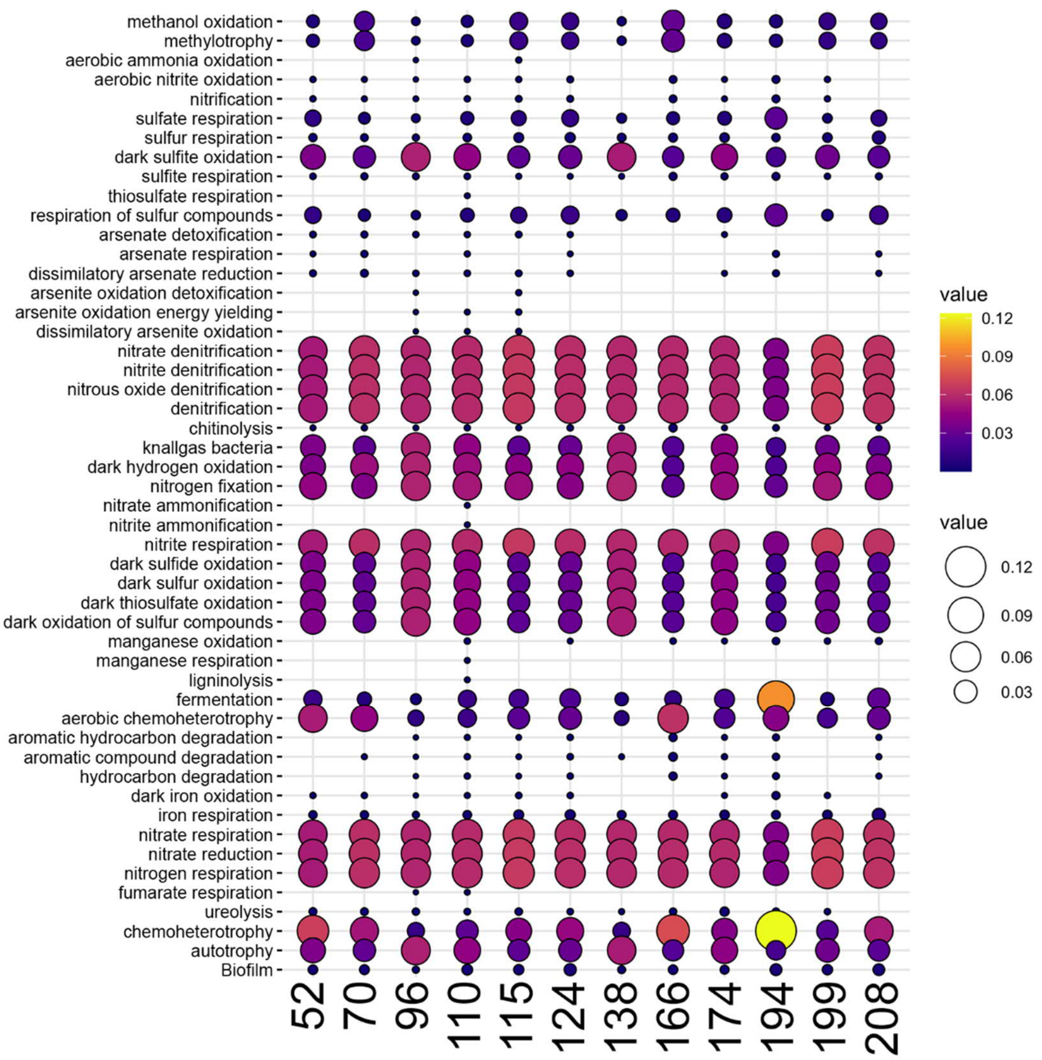

Fig. S2 Metabolic predictions of bacteria in BR 2 by sampling day according to FAPROTAX (Louca et al., 2016; Bomberg, 2020).

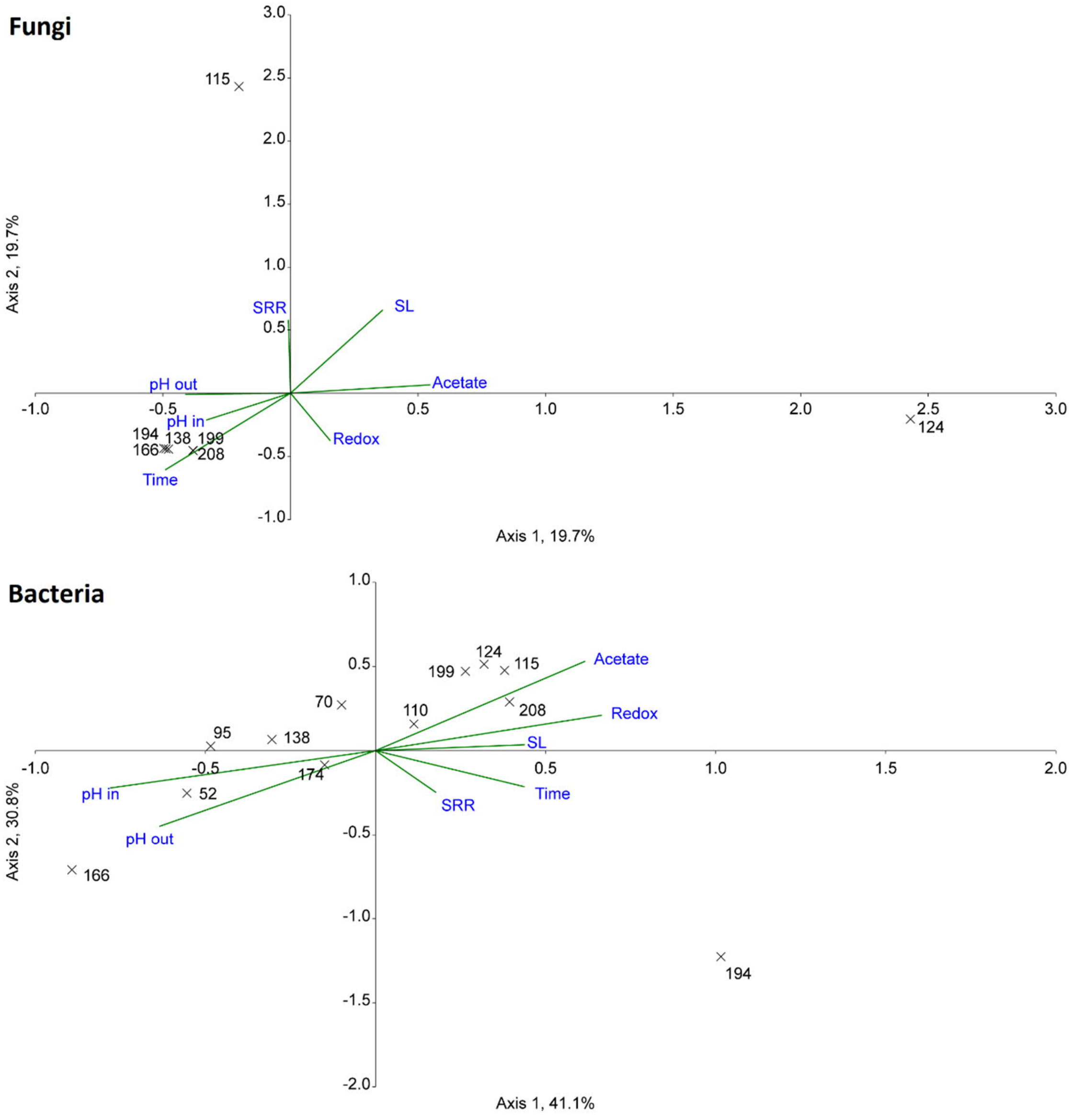

Fig. S3 CCA analyses of BR 2 microbial data against the measured parameters, axes show percentage of variance. Data point labels refer to sampling dates. SL = sulfate loading rate, SRR = sulfate removal rate.

Table S1 Number of sequences, OTUs, Chao1 and Shannon indices in archaea in each BSR bioreactor sample. Chao1 and Shannon's diversity indices are not included for these samples as only few had a sequence count of at least 200.

Bioreactor	Day	Sequence counts	OTUs	Chao1	Shannon
BR 1 A	49	93	7		
	57	78	6		
	92	10	3		
	135	38	7		
	251	669	9		
	315	2062	17		
	319	1241	18		
	322	1562	19		
	333	3540	26		
BR 1 B	49	157	4		
	57	135	5		
	92	31	2		
	135	131	4		
	251	78	4		
	315	640	12		
	322	477	12		
	329	140	6		
	333	27	4		
BR 2	52	19	3		
	70	13	3		
	95	10	3		
	110	13	2		
	115	1	1		
	124	0	0		
	138	9	4		
	166	371	7		
	174	37	2		
	194	11	2		
	199	52	7		
	208	40	7		

Table S2 Number of sequences, OTUs, Chao1 and Shannon indices in fungi in each BSR bioreactor sample. Chao1 and Shannon's diversity indices are calculated only when the sequence count is at least 467.

Bioreactor	Day	Sequence counts	OTUs	Chao1	Shannon
BR 1 A	49	3322	43	46	2.0
	57	467	20	21	1.0
	92	1219	29	31	1.9
	135	1556	30	39	1.4
	251	3566	40	45	1.3
	315	1677	48	57	1.9
	319	3329	24	29	0.2
	322	1049	57	78	2.1
	333	2940	39	54	0.6
BR 1 B	49	682	30	39	2.5
	57	339			
	92	478	28	34	2.3
	135	70			
	251	833	24	26	1.4
	315	776	32	37	1.7
	322	1187	42	68	1.4
	329	811	14	19	0.7
	333	975	23	37	0.6
BR 2	52	243			
	70	26			
	95	354			
	110	103			
	115	6744	35	40	0.2
	124	6114	22	23	0.2
	138	4810	43	45	2.0
	166	1211	20	21	1.6
	174	4			
	194	4108	26	40	1.3
	199	5995	31	34	0.2
	208	13053	45	46	1.0

Table S3 Number of sequences, OTUs, Chao1 and Shannon's diversity indices in bacteria in each BSR bioreactor sample.

Bioreactor	Day	Sequence counts	OTUs	Chao1	Shannon
BR 1 A	49	7026	159	238	2.5
	57	5669	114	165	2.3
	92	9576	224	337	3.1
	135	8181	154	226	2.1
	251	4204	159	266	2.5
	315	6382	173	229	2.6
	319	8207	184	389	2.8
	322	4810	116	187	2.2
	333	6644	162	218	2.1
BR 1 B	49	6104	148	298	2.8
	57	1201	66	105	2.7
	92	4747	130	169	2.5
	135	6481	144	212	2.0
	251	3001	123	196	2.5
	315	8385	175	254	2.7
	322	1683	85	148	2.3
	329	8506	199	352	2.3
	333	6075	136	189	1.7
BR 2	52	2448	109	148	2.5
	70	1717	98	156	2.5
	95	3513	88	121	1.4
	110	3681	136	240	2.3
	115	2719	118	161	2.5
	124	2206	120	200	2.6
	138	2873	91	107	1.6
	166	867	80	113	2.6
	174	1927	109	184	2.6
	194	2588	111	136	2.9
	199	3459	120	148	2.2
	208	1521	72	95	2.7
